# Адипомиокины у детей с конституционально-экзогенным ожирением

**DOI:** 10.14341/probl13250

**Published:** 2023-08-30

**Authors:** Ю. В. Бурмицкая, О. В. Васюкова, П. Л. Окороков, З. Т. Зураева, О. Б. Безлепкина

**Affiliations:** Национальный медицинский исследовательский центр эндокринологии; Национальный медицинский исследовательский центр эндокринологии; Национальный медицинский исследовательский центр эндокринологии; Национальный медицинский исследовательский центр эндокринологии; Национальный медицинский исследовательский центр эндокринологии

**Keywords:** миокины, адипомиокины, ожирение, дети, подростки, интерлейкин-6, миостатин, декорин, ирисин, биоимпедансометрия

## Abstract

ОБОСНОВАНИЕ. Адипомиокины синтезируются и секретируются в кровоток клетками как мышечной, так и жировой ткани. Они могут оказывать как отрицательное метаболическое воздействие, выступая в роли провоспалительных адипокинов при ожирении, так и положительное, повышаясь в ответ на физические нагрузки в виде миокинов.ЦЕЛЬ. Изучить особенности секреции адипомиокинов у детей с конституционально-экзогенным ожирением.МАТЕРИАЛЫ И МЕТОДЫ. В исследование включены 80 пациентов: 60 подростков 15 [13; 16] лет с конституционально-экзогенным ожирением, SDS индекса массы тела (ИМТ): 3,0 [2,6; 3,3], и 20 детей группы контроля 16 [15; 17] лет без избытка массы тела, SDS ИМТ: -0,3 [-1,25; 0,33]. Для определения уровня адипомиокинов использованы коммерческие наборы для иммуноферментного анализа. Композиционный состав тела оценивался методом биоимпедансного анализа (анализатор In Body 770, Южная Корея) утром, натощак. Статистическая обработка проводилась с применением STATISTICA v.12.0 (StatSoftInc., США). Результаты представлены в виде медианы (Ме) и квартилей (Q1; Q3), соответствующих 25 и 75 перцентилям. Критический уровень значимости (р) принимали равным <0,05.РЕЗУЛЬТАТЫ. Уровни интерлейкина-6 (ИЛ-6) и ирисина статистически значимо выше у подростков с ожирением по сравнению с группой контроля: 0,55 [0,226; 1,35] vs 0,202 [0,128; 0,652] пг/мл (р=0,041) и 11,16 [6,6; 22,76] vs 7,36 [6,48; 9,68] мкг/мл (р=0,043) соответственно. Концентрации ИЛ-6, миостатина и декорина повышаются при увеличении степени ожирения: I степень vs III: 0,226 [0,224; 0,398] vs 0,80 [0,36; 1,81] пг/мл (р=0,0197), 25,85 [21,53; 28,23] vs 31,41 [24,36; 35,06] нг/мл (р=0,03), 4065,3 [3244,9; 5245,5] vs 5322,5 [4199,8; 7702,4] пг/мл (р=0,0376) соответственно. У детей с ожирением уровни ИЛ-6 положительно коррелируют с ИМТ, SDS ИМТ и количеством жировой ткани, а миостатина — с ИМТ и SDS ИМТ. Концентрация ирисина в сыворотке крови значимо выше у девочек с ожирением, чем у мальчиков с ожирением и здоровых девочек. Пациенты с ожирением, по сравнению с худыми сверстниками, характеризуются статистически значимо большим содержанием как жировой, так и тощей массы. При прогрессировании ожирения отмечается статистически значимое повышение отношения жировой к тощей массе (I степень — 0,66 [0,56; 0,7], III — 0,78 [0,68; 0,98]; р=0,0073).ЗАКЛЮЧЕНИЕ. Пациенты с ожирением и нормальной массой тела имеют различные уровни адипомиокинов. Повышение уровня ИЛ-6 при прогрессировании ожирения напрямую связано с увеличением содержания жировой ткани. Требуется дальнейшее изучение особенностей секреции адипомиокинов, их взаимосвязи с композиционным составом тела и метаболическими осложнениями при ожирении.

## Abstract (en)

BACKGROUND: Adipomyokines are synthesized and secreted into the bloodstream by cells of both muscle and adipose tissue. They can have both a negative metabolic effect, acting as pro-inflammatory adipokines in obesity, and a positive one, increasing in response to physical exertion in the form of myokines.AIM: To study the features of adipocytokine secretion in children with constitutionally exogenous obesity.MATERIALS AND METHODS: The study included 80 patients: 60 adolescents aged 15 [13; 16] years with constitutionally exogenous obesity SDS BMI: 3.0 [2.6; 3.3] and 20 control group children aged 16 [15; 17] years without excess weight SDS BMI: -0.3 [-1.25; 0.33]. Commercial enzyme immunoassay kits were used to determine the level of adipomyokines. The compositional composition of the body was evaluated by bioimpedance analysis (InBody 770 analyzer, South Korea) in the morning, on an empty stomach. Statistical processing was carried out using STATISTICA v.12.0 (StatSoft Inc., USA). The results are presented in the form of median (Me) and quartiles (Q1; Q3) corresponding to 25 and 75 percentiles. The critical significance level (p) was assumed to be <0.05.RESULTS: Levels of IL-6 and irisin are statistically significantly higher in obese adolescents compared to the control group: 0.55 [0.226; 1.35] pg/ml vs 0.202 [0.128; 0.652] pg/ml (p=0.041) and 11.16 [6.6; 22.76] mcg/ml vs 7.36 [6.48; 9.68] mcg/ml (p=0.043), respectively. Concentrations of IL-6, myostatin and decorin increase with an increase in the degree of obesity: grade I vs III: 0.226 [0.224; 0.398] vs 0.80 [0.36; 1.81] pg/ml (p=0,0197), 25,85 [21,53; 28,23] vs 31.41 [24.36; 35.06] ng/ml (p=0.03), 4065.3 [3244.9; 5245.5] vs 5322.5 [4199.8; 7702.4] pg/ml (p=0.0376), respectively. In obese children, IL-6 levels positively correlate with BMI, SDS BMI and the amount of adipose tissue, and myostatin — with BMI and SDS BMI. The concentration of irisin in the blood serum is significantly higher in obese girls than in obese boys and healthy girls. Obese patients, compared with lean peers, are characterized by a statistically significantly higher content of both fat and lean mass. With the progression of obesity, there is a statistically significant increase in the ratio of fat to lean mass (I degree — 0.66 [0.56; 0.7], III — 0.78 [0.68; 0.98] (p=0.0073).CONCLUSION: Patients with obesity and normal body weight have different levels of adipomyokines. An increase in the level of IL-6 with the progression of obesity is directly related to an increase in the content of adipose tissue. Further study of the features of adipocytokine secretion, their relationship with the features of the body composition and metabolic complications in obesity is required.

## ОБОСНОВАНИЕ

Скелетные мышцы представляют собой не только активную часть опорно-двигательного аппарата, но и рассматриваются как эндокринный орган, который синтезирует и секретирует специфические белки — миокины — во время физических нагрузок, оказывая аутокринные, паракринные и эндокринные эффекты [1]. В настоящее время открыты и активно изучаются сотни таких белков: ирисин, миостатин, декорин, интерлейкины (ИЛ-6, -7, -10, -15 и др.). Они опосредуют положительные эффекты, связанные с физическими упражнениями, участвуя в процессах пролиферации, дифференцировки и регенерации мышечной ткани, энергетическом обмене в скелетной мускулатуре, а также взаимодействуя с другими метаболически активными органами: жировой тканью (ЖТ), печенью, поджелудочной железой, костной тканью [2]. С другой стороны, при малоподвижном образе жизни и избыточной массе тела миокины активно синтезируются в адипоцитах и выступают уже в роли провоспалительных цитокинов — адипомиокинов. Регулярные физические упражнения профилактируют развитие и прогрессирование метаболических осложнений, а повышающиеся в ответ на физическую нагрузку миокины могут нивелировать провоспалительные эффекты адипокинов.

Таким образом, перед нами открывается двойственная роль миокинов. С одной стороны, при ожирении и низком уровне физической активности адипомиокины накапливаются в ЖТ и секретируются в системный кровоток, оказывая отрицательное метаболическое влияние. С другой стороны, при сокращении мышечных волокон во время физических нагрузок они активно синтезируются и секретируются в кровяное русло, оказывая положительные эффекты как на уровне мышечной ткани, так и системно, снижая уровень низкоактивного метаболического воспаления.

Особенности секреции адипомиокинов, их взаимосвязь с показателями композиционного состава тела у детей и подростков с ожирением изучены недостаточно.

## ЦЕЛЬ ИССЛЕДОВАНИЯ

Изучить особенности секреции адипомиокинов у детей с конституционально-экзогенным ожирением.

## МАТЕРИАЛЫ И МЕТОДЫ

## Дизайн исследования

Проведено обсервационное одноцентровое одномоментное выборочное контролируемое исследование.

## Критерии соответствия

В исследование включены 60 пациентов (28 мальчиков и 32 девочки) от 12 до 17,9 года с конституционально-экзогенным ожирением, SDS ИМТ≥+2,0 (SDS — от англ. standart deviation score — коэффициент стандартного отклонения; ИМТ — индекс массы тела), с половым развитием по Таннеру 4–5 стадии. Группу контроля составили 20 здоровых подростков от 12 до 17,9 года, без избытка массы тела, SDS ИМТ от -2 до +1, с половым развитием по Таннеру 4–5 стадии.

Критериями исключения из исследования были получение медикаментозной терапии ожирения на момент обследования, наличие других форм ожирения (гипоталамическое, синдромальное и др.), тяжелых сопутствующих заболеваний (ортопедическая патология, заболевание легочной системы, некомпенсированная артериальная гипертензия, психические расстройства), патологии надпочечников, щитовидной железы (в состоянии гипотиреоза, тиреотоксикоза), сахарного диабета 1 и 2 типа.

## Место и время проведения исследования

Место проведения. Исследование проводилось в ФГБУ «НМИЦ эндокринологии» Минздрава России.

Время исследования. Включение пациентов в исследование проведено с января по октябрь 2022 г. Образцы сывороток исследовались одномоментно после сбора всего биологического материала.

## Описание медицинского вмешательства

Антропометрические измерения включали: измерение роста, массы тела, расчет ИМТ. ИМТ оценивался по нормативам для конкретного возраста и пола и представлен в виде числа стандартных отклонений от среднего (SDS). Оценка полового развития проводилась по классификации Tanner. Всем пациентам выполнена оценка композиционного состава тела. Исследование уровней миокинов в сыворотке крови проводилось утром натощак (в покое).

## Исходы исследования

Основной исход исследования: определены сывороточные уровни адипомиокинов — ИЛ-6, миостатина, декорина, ирисина в состоянии покоя утром натощак у пациентов с ожирением и без избытка массы тела.

Дополнительные исходы исследования: определены и изучены особенности композиционного состава тела: содержание ЖТ (%, кг), тощей массы (ТМ, кг), скелетно-мышечной массы (СММ, %, кг), соотношение жировой массы (ЖМ) к ТМ (ЖМ/ТМ).

## Методы регистрации исходов

Значение SDS ИМТ от 2,0 до 2,5 определяли как ожирение I степени, SDS ИМТ от 2,6 до 3,0 — II степени, SDS ИМТ от 3,1 до 3,9 — III степени, SDS ИМТ от -2 до +1 — как нормальную массу тела [3].

Лабораторные исследования проводились в клинико-диагностической лаборатории ФГБУ «НМИЦ эндокринологии» Минздрава России. Концентрация миокинов в сыворотке крови определялась наборами для иммуноферментного анализа: ирисин — Biovendor, ИЛ-6 –Bender Medsystems, декорин — Aviscera Bioscience, миостатин — ELISA kit Immundiagnostik AG.

Оценка композиционного состава тела проводилась методом биоимпедансного анализа (анализатор In Body 770, Южная Корея) утром, натощак. Показатель ЖМ/ТМ более 90 перцентиля для данного пола и возраста использован в качестве критерия «саркопенического ожирения» у детей [4].

## Этическая экспертиза

Протокол исследования одобрен локальным этическим комитетом при ФГБУ «Национальный медицинский исследовательский центр эндокринологии» Минздрава России (выписка из протокола №2 от 12.02.2020 г.). Родители всех включенных пациентов подписали добровольное информированное согласие на участие в исследовании.

## Статистический анализ

Статистическая обработка данных проводилась с использованием программ Excel 2016 (Microsoft, USA), Statistica (версии 12.0, StatSoftInc., США). Результаты представлены в виде медианы (Ме) и квартилей (Q1; Q3). Для оценки достоверности различий между изучаемыми подгруппами использовался критерий Манна–Уитни и дисперсионный анализ Краскела–Уоллеса. Корреляционный анализ проводился с использованием критерия Спирмена. Критический уровень значимости различий принимали ≤0,05.

## РЕЗУЛЬТАТЫ

## Клиническая характеристика обследованных пациентов

В исследование включены 80 пациентов: 60 подростков с конституционально-экзогенным ожирением (28 мальчиков и 32 девочки, 15 [ 13; 16] лет) и 20 детей группы контроля, 8 мальчиков и 12 девочек, 16 [ 15; 17] лет с нормальной массой тела.

Конституционально-экзогенное ожирение I степени было у 14 пациентов (23%), 11 девочек и 3 мальчиков, II степени — у 16 подростков (27%), 9 девочек и 7 мальчиков, III степени — у 30 детей (50%), 17 мальчиков и 13 девочек.

Антропометрические показатели и параметры композиционного состава тела представлены в таблице 1. Пациенты с ожирением статистически значимо отличались от группы контроля по массе тела, ИМТ, SDS ИМТ и по исследуемым показателям композиционного состава тела. При этом количество ЖМ у детей с ожирением более чем в три раза превышало таковую у здоровых сверстников, а содержание ТМ было на четверть выше у пациентов с ожирением по сравнению с группой контроля. Соответствующие различия отражает соотношение ЖМ/ТМ, которое у детей с ожирением оказалось статистически значимо выше, чем у пациентов группы контроля: 0,73 [ 0,61; 0,85] и 0,3 [ 0,23; 0,39] соответственно (р<0,0001).

**Table:** Таблица 1. Антропометрические параметры и показатели композиционного состава тела исследуемых групп Данные представлены в виде медианы с интерквартильным интервалом Ме [ Q1; Q3].SDS — стандартное отклонение от среднего.

	Ожирение (n=60)	Группа контроля (n=20)	Р
Рост, см	169 [ 165,2; 176,2]	165,3 [ 161,9; 173,0]	>0,05
SDS роста	0,64 [ 0,29; 1,7]	-0,12 [ 0,29; 1,7]	>0,05
Масса тела, кг	94 [ 84,5; 106,0]	55,5 [ 50,3; 61,0]	<0,0001
ИМТ	33,1 [ 30,5; 36,6]	19,1 [ 17,4; 20,9]	<0,0001
SDS ИМТ	3,0 [ 2,6; 3,3]	-0,3 [ -1,25; 0,33]	<0,0001
Стадия полового развития, Таннер	4–5	4–5	>0,05
% ЖТ	40,8 [ 36,5; 44,3]	22,2 [ 17,8; 31,6]	<0,0001
ЖТ, кг	36.8 [ 30,9; 46,0]	11,3 [ 8,9; 16,7]	<0,0001
ТМ, кг	49,8 [ 44,4; 59,5]	39,3 [ 33,4; 42,1]	<0,0001
Безжировая масса, кг	53,45 [ 47,9; 63,4]	41,8 [ 35,5; 44,7]	<0,0001
СММ, кг	29,8 [ 26,4; 36,1]	22,5 [ 19,2; 24,3]	<0,0001
ЖМ/ТМ	0,73 [ 0,61; 0,85]	0,3 [ 0,23; 0,39]	<0,0001

В то же время при возрастании степени ожирения, помимо увеличения количества ЖМ, отмечается повышение содержания безжировой, ТМ и СММ (табл. 2). Однако ввиду диспропорционального изменения композиционного состава тела при ожирении у пациентов с III степенью ожирения по сравнению с I степенью количество ЖМ более чем в 2,5 раза превышает количество ТМ. Данная особенность отчетливо прослеживается при анализе индекса соотношения ЖМ/ТМ при возрастании степени ожирения. Так, ЖМ/ТМ при III степени составило 0,78 [ 0,68; 0,98], что статистически значимо выше, чем при I степени 0,66 [ 0,56; 0,7] (р=0,0073). Наряду с этим, соотношение ЖМ/ТМ более 90 перцентиля выявлено у 94% пациентов с ожирением, что характеризует крайне высокую частоту «саркопенического ожирения».

**Table:** Таблица 2. Антропометрические параметры и показатели композиционного состава тела подростков с разной степенью ожирения

	I степень (n=14)	II степень (n=16)	III степень (n=30)	Р
Рост, см	169,0 [ 167,7;172,1]	167,2 [ 163,6; 172,9]	172,0 [ 164,1; 177,0]	I–II>0,05 I–III>0,05 II–III>0,05
Масса тела, кг	84,5 [ 78,3; 88,0]	84,8 [ 80,0; 96,5]	105 [ 94,7; 116,0]	I–II>0,05 I–III<0,0001 II–III<0,0001
ИМТ	29,1 [ 28,3; 29,6]	30,4 [ 28,6; 32,6]	35,7 [ 33,6; 40,1]	I–II>0,05 I–III<0,0001 II–III<0,0001
SDS ИМТ	2,24 [ 2,1; 2,37]	2,78 [ 2,6; 2,83]	3,3 [ 3,07; 3,7]	I–II>0,05 I–III<0,0001 II–III<0,0001
% ЖТ	38,2 [ 34,5; 39,7]	40,5 [ 34,2; 44,0]	42,4 [ 40,1; 47,1]	I–II>0,05 I–III=0,0023 II–III=0,06
ЖТ, кг	30,1 [ 28,0; 31,3]	32,6 [ 30,2; 38,8]	45,6 [ 38,5; 50,3]	I–II>0,05 I–III<0,0001 II–III<0,0001
ТМ, кг	45,1 [ 43,8; 53,1]	45,3 [ 42,7; 55,8]	51,5 [ 47,3; 63,7]	I–II>0,05 I–III=0,025 II–III=0,06
Безжировая масса, кг	48,0 [ 46,6; 56,6]	46,5 [ 45,2; 59,3]	57,9 [ 51,9; 67,7]	I–II>0,05 I–III=0,004 II–III=0,009
СММ, кг	26,5 [ 25,6; 31,6]	26,6 [ 25,0; 33,6]	31,9 [ 28,3; 39,6]	I–II>0,05 I–III=0,006 II–III=0,027
СММ, %	33,3 [ 32,3; 35,9]	32,2 [ 30,8; 36,8]	32,2 [ 29,4; 34,5]	I–II>0,05 I–III>0,05 II–III>0,05
ЖМ/ТМ	0,66 [ 0,56; 0,7]	0,72 [ 0,55; 0,83]	0,78 [ 0,68; 0,98]	I–II>0,05 I–III=0,0073 II–III>0,05

Интерлейкин-6

Уровень ИЛ-6 у подростков с ожирением составил 0,55 [ 0,226; 1,35] пг/мл и был статистически значимо выше, чем у детей группы контроля — 0,202 [ 0,128; 0,652] пг/мл (р=0,041) (рис. 1).

**Figure:**
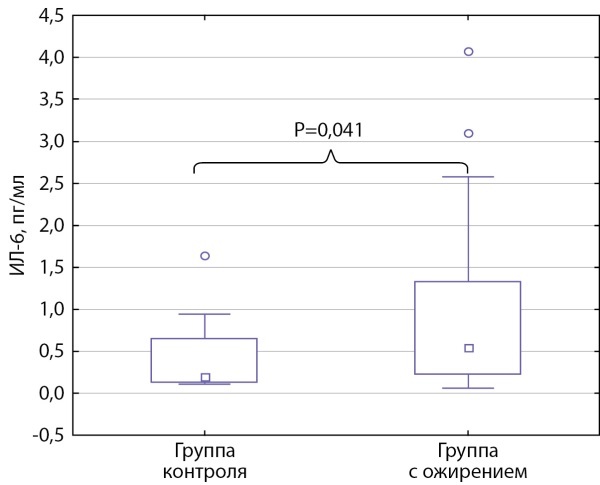
Рисунок 1. Уровень ИЛ-6 у подростков с ожирением и группы контроля.Данные представлены в виде медианы с интерквартильным интервалом Ме [ Q1; Q3]. Использовался критерий Манна–Уитни.

Выявлено повышение концентрации ИЛ-6 при увеличении степени ожирения. У детей с I степенью ожирения уровень ИЛ-6 составил 0,226 [ 0,224; 0,398], со II — 0,516 [ 0,426; 0,794], с III — 0,80 [ 0,36; 1,81] пг/мл. Уровень ИЛ-6 при II и III степени ожирения был статистически значимо выше, чем при I степени (р=0,0425 и 0,0197 соответственно) и у детей группы контроля (р=0,0446 и 0,0049 соответственно) (рис. 2).

**Figure:**
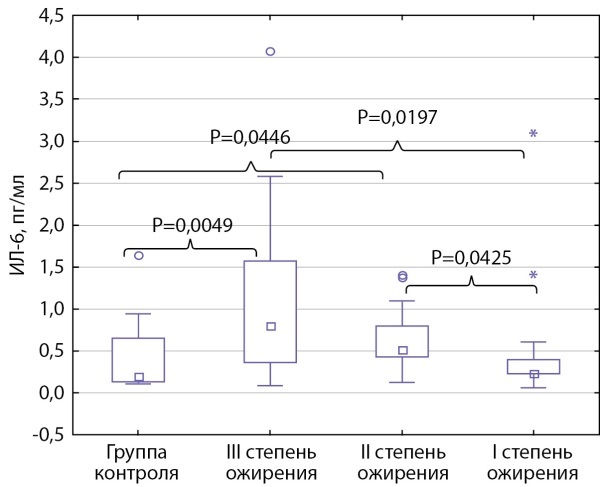
Рисунок 2. Уровень ИЛ-6 у подростков с разной степенью ожирения и группы контроля.Данные представлены в виде медианы с интерквартильным интервалом Ме [ Q1; Q3]. Использовался дисперсионный анализ Краскела–Уоллиса.

У пациентов с ожирением выявлена положительная корреляционная связь между уровнем ИЛ-6 и массой тела (r=0,278), ИМТ (r=0,364), SDS ИМТ (r=0,315), а также количеством ЖТ (r=0,354). В группе контроля взаимосвязи ИЛ-6 с антропометрическими показателями получено не было.

Таким образом, более высокий уровень ИЛ-6 у пациентов с ожирением по сравнению с группой контроля, а также его увеличение при прогрессировании ожирения связано с повышением количества ЖТ.

Миостатин

Уровни миостатина статистически значимо не различались у детей с ожирением и группой контроля — 28,95 [ 22,94; 33,56] и 29,43 [ 27,08; 31,19] нг/мл соответственно.

Однако выявлены изменения уровня миостатина в зависимости от степени ожирения. При I степени его концентрация в сыворотке крови составила 25,85 [ 21,53; 28,23], при II — 30,58 [ 23,94; 31,65] и максимальная концентрация отмечена при III степени — 31,41 [ 24,36; 35,06] нг/мл. Статистически значимые различия выявлены между I и III степенью ожирения (р=0,03). У пациентов с ожирением установлена положительная корреляционная связь между уровнем миостатина и ИМТ (r=0,39), SDS ИМТ (r=0,26), что объясняет полученные результаты дисперсионного анализа. У пациентов группы контроля подобной взаимосвязи не выявлено.

Учитывая, что миостатин является ингибитором роста мышечной ткани, можно предположить, что его уровень повышается при увеличении степени ожирения ввиду уменьшения количества ТМ относительно ЖМ, что косвенно проявляется высокой частотой «саркопенического ожирения» в исследуемой группе пациентов.

Декорин

У подростков с ожирением уровень декорина составил 4743 [ 3996; 7609] пг/мл и был ниже, чем у здоровых детей, — 6127 [ 4706; 8182] пг/мл, однако статистически значимой разницы не выявлено (р=0,122).

Вместе с тем отмечено повышение уровня декорина с увеличением степени ожирения. При I степени его концентрация составила 4065,3 [ 3244,9; 5245,5] пг/мл, при II — 4608,8 [ 3890; 7881,8] пг/мл, при III степени — 5322,5 [ 4199,8; 7702,4] пг/мл. Статистически значимые различия выявлены между I и III степенью ожирения (р=0,0376), а также между группой контроля и пациентами с ожирением I степени (р=0,0248). Несмотря на это, корреляционных связей с анализируемыми антропометрическими показателями и параметрами композиционного состава тела не выявлено. Вероятно, что у детей с ожирением в связи с непропорциональным увеличением жировой массы относительно тощей уровень декорина ниже, чем у подростков группы контроля. А увеличение декорина при прогрессировании ожирения может быть связано с повышением абсолютных значений тощей массы.

Ирисин

Концентрация ирисина была статистически значимо выше у подростков с ожирением — 11,16 [ 6,6; 22,76] мкг/мл по сравнению с контрольной группой — 7,36 [ 6,48; 9,68] мкг/мл (р=0,043).

Максимальный уровень ирисина отмечен при I степени ожирения — 22,52 [ 6,68; 35,56] мкг/мл, средний при III степени — 11,16 [ 6,24; 19,34] мкг/мл, минимальный при II — 9,36 [ 7,44; 22,27] мкг/мл. Статистически значимых различий между степенями ожирения, а также по сравнению с группой контроля выявлено не было. Однако у девочек с ожирением отмечался статически значимо более высокий уровень ирисина — 14,52 [ 6,68; 33,32] мкг/мл, чем у мальчиков — 7,76 [ 5,72; 14,74] мкг/мл (р=0,029236) и у девочек группы контроля — 7,44 [ 6,68; 9,68] (р=0,043).

Ввиду того, что при I степени ожирения количество ТМ/ЖМ больше, чем при II и III степени, уровень ирисина максимален при I степени. Поскольку количество ТМ и СММ значимо больше у детей с ожирением, чем у подростков с нормальной массой тела, то уровень ирисина соответственно выше.

## ОБСУЖДЕНИЕ

В проведенном исследовании у подростков с ожирением уровень ИЛ-6 был в два раза выше, чем у группы контроля. Получено статистически значимое повышение уровня ИЛ-6 при увеличении степени ожирения. Установлена положительна корреляционная связь между ИЛ-6 и массой тела, ИМТ, SDS ИМТ, а также количеством ЖТ.

Согласно полученным результатам, прогрессирование ожирения сопровождается выраженными изменениями композиционного состава тела, а именно диспропорциональным увеличением содержания жировой и тощей массы, что характеризуется повышением индекса ЖМ/ТМ и свидетельствует о развитии «саркопенического ожирения». Известно, что данное состояние ассоциировано с высоким риском метаболических осложнений и преждевременной смертности [5]. Результаты проведенного исследования согласуются с ранее полученными данными об особенностях композиционного состава тела у детей с морбидным ожирением [6], демонстрирующих высокую распространенность «саркопенического ожирения» (88,68%) у детей с конституционально-экзогенным ожирением [7]. Наряду с этим, диспропорциональное увеличение количества ЖМ относительно ТМ и снижение содержания ТМ относительно ЖМ при прогрессировании ожирения, предположительно, может быть связано с изменением миокинового профиля.

Более высокий уровень ИЛ-6 у пациентов с ожирением, по сравнению с пациентами без избытка массы тела, отмечен в многочисленных исследованиях как у взрослых, так и у детей [8–11], что согласуется с результатами нашей работы.

В работе D.M.E. El-Mikkawy и соавт. у взрослых пациентов (36,40±7,26 года, n=60) с ожирением и избыточной массой тела уровень ИЛ-6 был статистически значимо выше, чем у лиц с нормальным весом (р=0,0001) и положительно коррелируется с ИМТ [12]. Схожие результаты получены и в других исследованиях на взрослых пациентах [13–15].

Таким образом, полученные результаты согласуются с общей тенденцией более высокого уровня ИЛ-6 у пациентов с ожирением, который в состоянии покоя преимущественно секретируется ЖТ, выступая в роли провоспалительного цитокина и маркера низкоактивного системного воспаления.

В нашем исследовании выявлены статистически значимое повышение уровня миостатина при возрастании степени ожирения, а также положительные корреляционные связи между миостатином и ИМТ, SDS ИМТ. Однако у пациентов группы контроля уровень миостатина был сопоставим с общей группой подростков с ожирением.

В литературе отмечаются противоречивые данные относительно уровня миостатина при ожирении и его взаимосвязи с антропометрическими и биохимическими параметрами, а исследования у детей немногочисленны.

В статье D.S Hittel и соавт. [16] при ожирении отмечается повышение уровня миостатина как в сыворотке крови, так и мРНК миостатина в мышечной ткани, кроме того, его концентрация снижается после значимой потери массы тела (после бариатрической операции) [17][18].

В работе S. Kern-Matschilles и соавт. у пациентов с ожирением отмечены положительные корреляционные связи между уровнем миостатина и ИМТ (r=0,235), процентным содержанием ЖТ (r=0,166), окружностью талии (r=0,206).

В работе M. Amor и соавт. [19] концентрация миостатина в крови была выше у пациентов с ожирением по сравнению с лицами без избытка массы тела, в то время как экспрессия миостатина в ЖТ между ними не отличалась. В работе F. Wang и соавт. [20] уровень миостатина был выше у пациентов с сахарным диабетом (СД) 2 типа и избыточной массой тела по сравнению со здоровыми пациентами (р<0,001). В обоих исследованиях была также выявлена положительная корреляционная связь с индексом инсулинорезистентности — НОМА.

В исследовании A. Efthymiadou и соавт. [21] у детей с СД 1 типа без избытка массы тела уровень миостатина был статистически значимо выше по сравнению со здоровыми детьми (р<0,0001). При этом уровень миостатина не коррелировал с ИМТ и уровнем гликированного гемоглобина.

В работе M. Baumgartner и соавт. [22], в которой исследовали уровень миостатина у детей (9–19 лет, 13,6±2,7 года) с ожирением (ИМТ выше 97-го процентиля; n=108) без контрольной группы, также не было выявлено корреляции с антропометрическими параметрами, но была установлена положительная взаимосвязь с уровнем инсулина и индексом НОМА (r=0,26, r=0,24 соответственно).

Таким образом, в большинстве статей отмечен более высокий уровень миостатина у взрослых пациентов с ожирением, его взаимосвязь с ИМТ и показателями инсулинорезистентности. Напротив, у детей зависимость уровня миостатина от массы тела неоднозначна, но прослеживается его взаимосвязь с показателями углеводного обмена. Требуется дальнейшее изучение особенностей секреции миостатина у детей.

В нашем исследовании выявлена тенденция к более низкому уровню декорина у пациентов с ожирением по сравнению с детьми группы контроля. Несмотря на это, обнаружено статистически значимое повышение концентрации декорина с возрастанием степени ожирения. Корреляционных связей с исследуемыми антропометрическими показателями не выявлено.

Исследования уровня декорина немногочисленны, большинство работ посвящено изучению декорина при физических нагрузках у взрослых. Статьи, включающие пациентов детского возраста, в литературе не найдены.

В одном из крупных исследований (n=286) K. Bolton и соавт. [24] исследовали экспрессию декорина в ЖТ, а также уровень декорина в крови у пациентов с СД 2 типа (ИМТ 35,9±3,0) и нормогликемией (ИМТ 25,3±0,7). Экспрессия мРНК декорина была выше в висцеральной, чем подкожно-жировой клетчатке в обеих группах, но преобладала у пациентов с ожирением и СД 2 типа. Уровень декорина в крови был выше на 12% у лиц с СД 2 типа в сравнении с пациентами с нормогликемией и нормальной массой тела (р=0,049).

Необходимы дальнейшие исследования особенностей секреции декорина у детей.

В проведенном исследовании выявлен статистически значимо более высокий уровень ирисина у детей с ожирением по сравнению с группой контроля. Несмотря на это, корреляционных связей с антропометрическими показателями и параметрами композиционного состава тела выявлено не было. Уровень ирисина у девочек с ожирением был статистически значимо выше, чем у мальчиков с ожирением и девочек с нормальной массой тела, при этом у подростков группы контроля концентрация ирисина была сопоставимой.

Подобные результаты были получены в работе B. Palacios-González и соавт. (n=85), в которой уровень ирисина был выше у детей с ожирением, чем у детей с нормальной и избыточной массой тела (р≤0,001) [25]. Однако в данном исследовании была выявлена положительная корреляционная связь между концентрацией ирисина и ИМТ (r=0,42), но гендерных различий не отмечалось.

Полученные нами результаты также согласуются с исследованием G. Çatlı и соавт., в котором отмечен более высокий уровень ирисина у детей с ожирением (n=36), чем у детей с нормальной массой тела (n=30) (р=0,024) [26]. При этом корреляционных связей с антропометрическими параметрами выявлено не было. В работе S. Blüher и соавт. у детей с ожирением (7–18 лет, n=65) также не отмечено взаимосвязи ирисина с массой тела, ИМТ, SDS ИМТ [27].

В статье Han Byul Jang и соавт. [28] уровень ирисина у подростков (12–15 лет) с ожирением (n=248) был статистически значимо выше (р<0,0001), чем у детей с нормальной массой тела (n=370), но были выявлены положительные корреляционные связи с ИМТ, SDS ИМТ, окружностью талии, количеством ЖТ. Однако гендерных различий у детей с ожирением установлено не было, а у девочек с нормальной массой тела уровень ирисина был значимо выше (р=0,006), чем у мальчиков.

В работе D. Löffler и соавт. (n=105) у девочек без избытка массы тела уровень ирисина был выше, чем у мальчиков (р=0,03), а у детей с ожирением гендерных различий не было выявлено [29]. Корреляционных связей с антропометрическими показателями, композиционным составом тела у детей выявлено не было. Напротив, у взрослых женщин с ожирением концентрация ирисина была статистически значимо выше, чем у женщин с нормальной массой тела (р=0,03), а у мужчин как с ожирением, так и с нормальной массой тела концентрация ирисина была выше, чем у женщин (р=0,03, р=0,04 соответственно). При этом у взрослых выявлена взаимосвязь с ИМТ, окружностью талии и активной клеточной массой.

Большинство работ свидетельствует о более высоком уровне ирисина у пациентов с ожирением, чем с нормальной и экстремально низкой массой тела как у детей, так и взрослых [30–32], что согласуется с полученными нами результатами. Однако остаются противоречивыми взаимосвязь ирисина с массой тела, ИМТ (SDS ИМТ), а также ассоциация ирисина с жировой или мышечной массой. Помимо этого, особенности композиционного состава тела у детей и взрослых по-разному влияют на уровень ирисина. Таким образом, требуется дальнейшее изучение особенностей секреции ирисина у детей.

## ОГРАНИЧЕНИЯ ИССЛЕДОВАНИЯ

Коллектив авторов допускает, что небольшой объем выборки группы контроля, а также наличие у большинства пациентов выраженного ожирения могли оказать определенное влияние на полученные результаты.

## ЗАКЛЮЧЕНИЕ

Секреция адипомиокинов у детей с ожирением и нормальной массой тела существенно различается. Увеличение степени ожирения сопровождается изменением концентрации адипомиокинов. Повышение уровня ИЛ-6 при прогрессировании ожирения напрямую связано с увеличением содержания ЖТ. Снижение уровня миостатина при увеличении степени ожирения, предположительно, обусловлено уменьшением количества ТМ относительно ЖМ. Необходимы дальнейшее изучение особенностей адипомиокинового профиля у детей и оценка их взаимосвязи с особенностями композиционного состава тела и метаболическими нарушениями, ассоциированными с ожирением.

## ДОПОЛНИТЕЛЬНАЯ ИНФОРМАЦИЯ

Источники финансирования. Публикация подготовлена в рамках госзадания «Новые подходы к персонифицированному лечению ожирения у детей на основе исследований энергетического обмена, функционального резерва бета-клеток, секреции адипокинов, миокинов и специфических шаперонов», регистрационный номер АААА-А20-120011790172-9.

Конфликт интересов. Коллектив авторов подтверждает отсутствие конфликта интересов по данному исследованию в ходе его проведения и на момент подачи рукописи данной статьи в редакцию, о котором следовало сообщить.

Участие авторов. Бурмицкая Ю.В. — существенный вклад в концепцию и дизайн исследования, получение, анализ данных и интерпретация результатов, написание статьи; Васюкова О.В., Окороков П.Л. — существенный вклад в концепцию и дизайн исследования, анализ результатов, внесение в рукопись существенной (важной) правки с целью повышения научной ценности статьи; Зураева З.Т. — получение результатов; Безлепкина О.Б. — внесение ценных замечаний. Все авторы одобрили финальную версию статьи перед публикацией, выразили согласие нести ответственность за все аспекты работы, подразумевающую надлежащее изучение и решение вопросов, связанных с точностью или добросовестностью любой части работы.
